# Editorial: Respiratory distress syndrome

**DOI:** 10.3389/fped.2022.1005998

**Published:** 2022-09-14

**Authors:** Ömer Erdeve, Kari D. Roberts, Peter A. Dargaville

**Affiliations:** ^1^Division of Neonatology, Department of Pediatrics, Ankara University School of Medicine, Ankara, Turkey; ^2^Division of Neonatology, Department of Pediatrics, University of Minnesota, Minneapolis, MN, United States; ^3^Menzies Institute for Medical Research, University of Tasmania, Hobart, TAS, Australia; ^4^Department of Paediatrics, Royal Hobart Hospital, Hobart, TAS, Australia

**Keywords:** respiratory distress syndrome, preterm, surfactant, polymorphism, vitamin D, altitude, sensitization

Therapeutic advances such as antenatal steroid (AS) use to enhance pulmonary maturity, delayed cord clamping and alveolar recruitment with continuous positive airway pressure in the delivery room, early rescue use of surfactant, and gentler ventilation strategies to minimize damage to the immature lung have significantly decreased respiratory distress syndrome (RDS) related morbidity and mortality in preterm infants. In addition, new advances in clinical use of third-generation synthetic surfactants, lung ultrasound as a diagnostic tool, precision medicine for decision of timing of surfactant treatment, use of surfactant as a therapeutic vector for mitigation of bronchopulmonary dysplasia (BPD) and widespread use of novel less-invasive surfactant administration techniques are examples of exciting new areas being explored (1–4).

The goal of this Frontiers in Pediatrics Research Topic was to provide up-to-date information on RDS for scientists and clinicians, and welcomed both clinical and experimental research articles on pathophysiology, diagnosis and management of RDS.

Understanding the role of genetic factors in the predisposition to disease is an area of medicine that has gained great interest in recent years. Associations between genetic variants of surfactant proteins and RDS have been reported but haplotypes of surfactant protein B gene (SFTPB) have not been studied. In the article by Mikolajcikova et al., the authors investigated haplotypes of common tagged single-nucleotide polymorphism and their association with RDS. In 149 preterm infants with no/ mild RDS compared to severe RDS, they found that the common haplotype GATGACA of the SFTPB gene can be protective against RDS while the haplotype GATGGCA may be a risk factor for RDS susceptibility. These finding contribute to better understanding of which preterm infants are at higher risk for severe RDS and may improve the ability to individualize targeted therapies.

Vitamin D is well-known for its active role in fetal bone metabolism, but it also plays a biological role in surfactant synthesis and normal lung development. Papalia et al. examined the impact of vitamin D status at birth on early respiratory outcomes of 109 infants born <29 weeks gestation. They found that low cord blood 25(OH)- vitamin D levels were associated with need for higher initial FiO_2_ levels, need for respiratory support on postnatal day 7, and increased rate of death or mechanical ventilation on postnatal day 7. These findings provide additional evidence for the important role of fetal vitamin D on lung development and the need to optomize maternal and neonatal vitamin D status.

In a retrospective cohort study, Li et al. examined the role of AS in pregnant women with gestational diabetes mellitus undergoing early term scheduled cesarean section. One thousand, one hundred sixty-five patients were divided based on the time interval from the first dose of AS administration to delivery: no steroids, <2, 2–7, and >7 days. Delivery within any time of AS administration was not related to decreased risk of RDS or transient tachypnea of the newborn, but was associated with an increased risk of neonatal hypoglycemia, with the highest risk in the group receiving steroids <2 days prior to delivery. These findings contibute to the recomendation that AS use should not extend beyond 34 weeks of gestation among women with diabetes.

You et al. designed a study to define risk factors associated with the development of RDS in infants with early onset sepsis (EOS) and to describe the clinical features. Two hundred and fifty infants diagnosed with EOS were divided into RDS and non-RDS groups in a retrospective manner for comparison. Lower birth weight, smaller gestational age, higher rates of premature rupture of membranes and preterm birth, lower Apgar score and lower serum albumin level were associated with increased risk of RDS. Logistic regression analysis identified low serum albumin level as independent risk factors for the development of RDS. Higher rates of complications, including pulmonary hemorrhage, intraventricular hemorrhage, septic shock, persistent pulmonary hypertension and BPD were found in the RDS group. Therefore, identifying early independent risk factors for the development of RDS in infants with EOS infants might be helpful to optomize outcomes.

Duan et al. investigated the therapeutic effect of surfactant replacement therapy at different altitudes. Since blood partial oxygen pressure, and hence the driving pressure for gas exchange in the lung, decreases with increasing altitude, it was postulated that altitude may play a role in RDS and response to therapy. The authors compared 337 infants with RDS at high-altitude (1,500–3,500 meters) and ultra-high-altitude (3,500–5,500 meters), and found that blood gas effect and mortality were better in the high-altitude group, but still effective at ultra-high altitudes. This is the first study to clarify the effect of surfactant replacement therapy on RDS in premature infants at different altitudes and is important to improving health care in these regions.

While surfactant therapy has dramatically improved outcomes for infants with RDS, little is known about the immunomodulatory effects of the medication. Yalaz et al. examined tracheal aspirates in 45 intubated preterm infants for cytokine and chemokine response following three different regimens of surfactant treatment (beractant 100 mg/kg, poractant-alfa 100 mg/kg and poractant-alfa 200 mg/kg). They found that different natural surfactant preparations have different immunomodulatory effects. These data provide insight into the quantitative changes in local and systemic immune mediators which may have effects on short and/or long-term outcomes.

The study by Shi et al. investigated the performance of the neonatal sequential organ failure assessment score in estimating mortality rate in RDS, with the aim of broadening its clinical application. In this study of 1,281 patients, patients with high scores had a higher mortality rate in comparison to patients with low scores. The authors concluded that the neonatal sequential organ failure assessment score was positively associated with the risk of mortality in RDS, therefore its use may aid physicians to identify high risk neonates quickly and accurately, and perform more aggressive intervention.

Taken together, the studies presented in this Research Topic add to our understanding of the risk factors and pathogenesis of RDS and the effects of exogenous surfactant treatment. We suggest that studies specifically on role of precision medicine in management of RDS and using surfactant as a therapeutic vector for mitigation of BPD may improve care of extreme premature infants in near future.

## Author contributions

ÖE, KR, and PD contributed to conception and design of the article. ÖE wrote the first draft of the manuscript. KR and PD wrote sections of the manuscript. All authors contributed to manuscript revision, read, and approved the submitted version.

## Conflict of interest

The authors declare that the research was conducted in the absence of any commercial or financial relationships that could be construed as a potential conflict of interest.

## Publisher's note

All claims expressed in this article are solely those of the authors and do not necessarily represent those of their affiliated organizations, or those of the publisher, the editors and the reviewers. Any product that may be evaluated in this article, or claim that may be made by its manufacturer, is not guaranteed or endorsed by the publisher.
